# Investigating the value of glucodensity analysis of continuous glucose monitoring data in type 1 diabetes: an exploratory analysis

**DOI:** 10.3389/fcdhc.2023.1244613

**Published:** 2023-09-11

**Authors:** Elvis Han Cui, Allison B. Goldfine, Michelle Quinlan, David A. James, Oleksandr Sverdlov

**Affiliations:** ^1^ Department of Biostatistics, University of California, Los Angeles, Los Angeles, CA, United States; ^2^ Division of Translational Medicine, Cardiometabolic Disease, Novartis Institutes for Biomedical Research, Cambridge, MA, United States; ^3^ Early Development Analytics, Novartis Pharmaceuticals Corporation, East Hanover, NJ, United States; ^4^ Methodology and Data Science, Novartis Pharmaceuticals Corporation, East Hanover, NJ, United States

**Keywords:** CGM, functional data analysis, glucodensity, pharmacodynamics, visualization

## Abstract

**Introduction:**

Continuous glucose monitoring (CGM) devices capture longitudinal data on interstitial glucose levels and are increasingly used to show the dynamics of diabetes metabolism. Given the complexity of CGM data, it is crucial to extract important patterns hidden in these data through efficient visualization and statistical analysis techniques.

**Methods:**

In this paper, we adopted the concept of glucodensity, and using a subset of data from an ongoing clinical trial in pediatric individuals and young adults with new-onset type 1 diabetes, we performed a cluster analysis of glucodensities. We assessed the differences among the identified clusters using analysis of variance (ANOVA) with respect to residual pancreatic beta-cell function and some standard CGM-derived parameters such as time in range, time above range, and time below range.

**Results:**

Distinct CGM data patterns were identified using cluster analysis based on glucodensities. Statistically significant differences were shown among the clusters with respect to baseline levels of pancreatic beta-cell function surrogate (C-peptide) and with respect to time in range and time above range.

**Discussion:**

Our findings provide supportive evidence for the value of glucodensity in the analysis of CGM data. Some challenges in the modeling of CGM data include unbalanced data structure, missing observations, and many known and unknown confounders, which speaks to the importance of--and provides opportunities for--taking an approach integrating clinical, statistical, and data science expertise in the analysis of these data.

## 1 Introduction

Type 1 diabetes (T1D) is a life-threatening autoimmune disease with no cure (1). Insulin replacement is lifesaving and treats symptoms of T1D, but does not alter disease progression. Glycated hemoglobin A_1c_ (HbA_1c_) is an established glycemic physiological biomarker, which is widely used for diagnosing, monitoring, and informing treatment decisions in the management of T1D. However, HbA_1c_ measurements may exhibit high variability and can be affected by various biological and analytic factors, which may complicate an accurate assessment of blood glucose and glycemic control in T1D (2).

Continuous glucose monitoring (CGM) is a technology increasingly used to capture the dynamics of metabolism in T1D (3). CGM provides a more complete assessment of glycemia than what is possible with intermittent evaluations by standard home blood glucose monitoring or HbA_1c_-based approaches. Modern CGM systems are wearable devices that can transmit glucose readings every 1 min–15 min to connected technologies, such as a smartphone, portable reading device, computer, thereby enabling patients, caregivers, and physicians to monitor glucose levels over time and make informed decisions in diabetes management. CGM is also an integral component of artificial pancreas systems—more advanced technologies that provide the means for an automatic insulin delivery at the right dose and at the appropriate times for the individual patient (4). The use of dashboard systems—software technologies integrating relevant clinical information, including CGM data into a unified display—hold promise for improving population-level management of T1D, especially in pediatric patients (5).

The structure of CGM data is complex. CGM measurements per individual represent high-frequency time series data capturing the dynamics of interstitial glucose concentrations. Although these data (in addition to HbA_1c_ measurements and other relevant biomarkers) can provide important information on diabetes metabolism, they require careful preprocessing and are challenging to analyze statistically (6, 7). As CGM data are frequently acquired under free-living conditions, they are subject to various sources of variability and confounding effects (8). Traditional statistical methods may not adequately capture the dynamic nature of the CGM data and the underlying patterns. Functional data analysis may provide a potentially useful alternative approach (9, 10). By considering the entire individual glucose trajectory as a functional unit and incorporating appropriate statistical models, functional data analysis may help researchers to characterize and compare glucose profiles, identify temporal patterns, and assess the impact of various factors on glucose dynamics (11, 12). Recently, Matabuena et al. (13) introduced a new functional representation of CGM data, termed “glucodensity”, with the corresponding statistical toolkit for analyzing glucodensities. They gave several important examples from both clinical practice and biomedical research, where the concept of glucodensity may be promising and provide a more accurate characterization of glucose metabolism than the more standard approaches. The predictive potential of glucodensity was also validated in the AEGIS study of long-term changes in glucose levels (14). Furthermore, the distributional presentation and analysis of high-frequency biomedical data can be useful in other areas of clinical research. For example, Matabuena et al. (15) showed the utility of this approach by deriving clinical phenotypes of older adults based on their accelerometry data and demonstrating the value of such phenotyping in predicting 5-year mortality and survival.

CGM data are now frequently collected in T1D clinical trials, commonly as a part of exploratory objectives and potentially as an outcome measure (16–19). In contrast to self-measured glucose using a fingerstick, CGM can provide continuous real-time measurement of glucose levels in an ambulatory setting, with the assessment of glycemic variability, comprehensive measurement of hyperglycemic and hypoglycemic exposure, and safety alerts for glycemic extremes. This extensive glycemic data should be of value for clinical trials of diabetes pharmacotherapies or strategies including nutrition and exercise. However, to date, the utility of CGM data acquired during a clinical trial has been somewhat limited, in part because the value of CGM-derived outcomes has not yet been fully recognized by regulators (i.e., the US Food and Drug Administration) or payers (i.e., insurance companies) as an indicator of safety or effectiveness.

This report provides insights on important statistical analysis issues with CGM data that arise in the context of randomized clinical trials of new investigational drugs for T1D. We highlight some challenges and outline ways to manage them using real data from an ongoing randomized, placebo-controlled phase 2a proof-of-concept study of an investigational medicinal product (IMP) in pediatric individuals and young adults with new-onset T1D. This study aimed to advance valuable approaches for clinical investigators, statisticians, and data scientists involved in the design and analysis of clinical trials collecting CGM data; however, the paper is not meant to provide comprehensive solutions.

## 2 Background and research questions

Consider a clinical research study with CGM data collection. As the actual IMP itself is not relevant to the CGM data, and because the study is ongoing, details of the IMP are not discussed. In brief, the trial is a non-confirmatory, randomized 2:1, placebo-controlled, investigator- and subject-blinded, parallel-arm, phase 2a trial to assess safety, tolerability, pharmacokinetics (PK), and the early efficacy of the IMP on the preservation of residual pancreatic beta-cell function in new-onset T1D in pediatric and young adult subjects. Eligible participants (newly diagnosed T1D patients, 12–21 years of age weighing between 30 kg and 125 kg) are enrolled into the trial within 8 weeks of the time of diagnosis based on the results of both screening and baseline visits [ClinicalTrials.gov Identifier: NCT04129528].

In this study, CGM data are collected as an exploratory objective for describing the dynamics of T1D metabolism. Study participants are provided with CGM devices (Dexcom G6) and supplies and asked to wear a CGM device for at least 3 consecutive days (10 days are preferred and feasible with the single sensor) at baseline prior to the first dose, at day 1 after receiving the first dose of the study drug, and at months 3, 6, 9, and 12 during the 1 year of treatment. Sufficient supplies are provided for continuous wear, which is encouraged. The acquired CGM data evaluate glycemic parameters, such as the mean glucose level, variability, time in the glycemic range, hyper and hypoglycemia, and duration, on the assessment days (20).

In this context, several important questions warrant investigation:

1. How can one perform an informative visualization of the longitudinal CGM data that would also be appealing from the clinician’s perspective? This is important as current visualizations are generally snapshots of 10 days to 14 days of data.
2. Based on the CGM data, can clinically meaningful subgroups be identified through the clustering of study participants?
3. Using the CGM data before and after randomization (i.e., “pretreatment” and “posttreatment”), can the treatment effect within a subject be quantified and can its significance be tested statistically (e.g., using an analog of a paired *t*-test)?
4. Is there a way to perform a statistical significance test comparing treatment effects (IMP vs. placebo) with respect to the CGM data, possibly accounting for some important covariates [e.g., an analog of a two-sample *t*-test or an analysis of covariance (ANCOVA)]?

To address the first two questions, we performed exploratory data analysis using a subset of data from the described phase 2a trial. These results are based on blinded data (i.e., the individual randomized treatment group information is unavailable), and they are presented in Section 4. To address the last two questions, some additional information on the individual treatment assignments and treatment periods is required. At this point, this information is unavailable because the study is still ongoing. Therefore, we present only the relevant data analysis strategy as part of the discussion in Section 5.

## 3 Materials and methods

**Table 1** below shows an example of the CGM data structure. The first column corresponds to the subject ID, the second column refers to the time at which the glucose levels were recorded, and the third column contains the values of the glucose levels (mg/dL). In our example, the glucose levels are recorded every 5 min. In all conducted analyses, we used all available valid CGM observations (i.e., observations not flagged as erroneous in the dataset) from the study participants, without the knowledge of their treatment assignments and without the knowledge of treatment periods within the subjects. Therefore, our analyses can be viewed as *unsupervised* learning.

**Table 1:** Example of a CGM data structure.

Subject ID	Time	Glucose level (mg/dL)
Subject 00000001	2022-01-01 10:55:00 UTC	155
Subject 00000001	2022-01-01 11:00:00 UTC	147
Subject 00000001	2022-01-01 11:05:00 UTC	138
Subject 00000002	2022-05-01 15:55:00 UTC	89
Subject 00000002	2022-05-01 16:00:00 UTC	97
Subject 00000002	2022-05-01 16:05:00 UTC	98
· · ·	· · ·	· · ·

The first column corresponds to the subject’s ID, the second column refers to the time at which the glucose levels were recorded, and the third column contains the values of the glucose levels (mg/dL).

### 3.1 Data visualization

Visualization of CGM data is of great importance to provide accessibility for the comprehension of the information in large datasets. Broll et al. (21) provided a comprehensive list of CGM-based metrics and an R package, *iglu*, for visualization purposes. We used the *iglu* package to obtain lasagna plots of average glucose levels per subject over the 24-h period. Also, we created plots of the available individual raw CGM data over 24 h, along with the mean ambulatory glucose profiles (AGPs) per subject. To visualize dynamic changes of glycemia within subjects, we created plots of individual time-in-range values compared with the study day.

### 3.2 Clustering using glucodensities

We adopt the concept of glucodensity (13) and demonstrate how one could benefit from a cluster analysis based on estimated glucodensities. The glucodensity is a probability density function describing the distribution of glucose levels over time. Formally, let Y(t) denote the glucose level measured by wearable devices at time t∈[0,T] . The glucodensity f(s) is defined as follows (13):


(1) $$f\left(s\right)\;=\;\frac{\partial }{\partial s}F(s),\text{ where }F\left(s\right)\;=\;\frac{1}{T}\int_{0}^{T}\text{Ι}\left(Y\left(t\right)\;\leq \;s\right)\text{d}t.$$

In Equation 1, Ι(·) stands for the indicator function. This formulation implicitly assumes that the glucose level Y(t) has a common density f(s) for all t∈[0,T] (22). A kernel density estimator (KDE) is applied for estimating f(s) . Let $y_{i}$ , i = 1,. . .,m be the realizations of Y(t) at time points $t_{1},.\;.\;.,t_{m}\;$ and $K_{h}\left(\cdot \right)$ be a kernel with bandwidth h . Then f(s) is estimated by:


(2) $$\hat{f}\left(s\right)\;=\;\frac{1}{m}\sum_{i\;=\;1}^{m}K_{h}(s\;-\;y_{i}).$$

Note that the measurements $y_{i}$ in Equation 2 are not independent. Theoretical justifications of the KDE for dependent data can be found in Hall et al. (23) and Bosq (22). Furthermore, Matabuena et al. (13) showed how the concept of the 2-Wasserstein distance (24) and energy-based methods (25, 26) can be applied to cluster-estimated glucodensities. We used the R package *biosensors.usc*, provided by Matabuena et al. (13), to estimate individual glucodensities and perform the corresponding cluster analysis.

To facilitate a clinical interpretation of the identified clusters, we performed the statistical comparisons of the clusters using analysis of variance (ANOVA) with respect to the baseline level of the C-peptide (an established biomarker of pancreatic beta-cell function) and with respect to conventional CGM-derived parameters such as the time in range (TIR), time above range, and time below range.

## 4 Results

### 4.1 Analysis dataset

Our analysis dataset included 30 subjects and 760,510 valid records in total. During the data preprocessing step, device error values were identified and flagged in the database (they represented < 5% of the observations) and discarded from the analysis. An assessment of potential bias due to temporary loss of data capture is outside the scope of the present paper. The overall time range from the first to the last observation for the 30 subjects was 4 days to 518 days, with a mean of 158.9 days and a median of 115.5 days. We defined the metric *coverage* as the number of days with valid observations covering at least 70% of each day, in line with the International Consensus on the Use of Continuous Glucose Monitoring (19, 20), which supports a minimum of approximately 70% of possible CGM readings over 14 days to enable an optimal glycemic assessment for real-time decision-making. Larger gaps in a 24-h period are generally considered insufficient for describing a daily profile. We note that our analysis differed in that we were assessing prespecified glycemic observation intervals to estimate glycemia over a broader interval of time, but we employed the same standard for a minimal definition of a complete day capture. In our data, the CGM coverage achieved by the 30 subjects ranged from 3 days to 468 days, with a mean of 84.7 days, a median of 45.5 days, a first quartile of 16.3 days, and a third quartile of 120 days.

### 4.2 Data visualization

**Figure 1:**
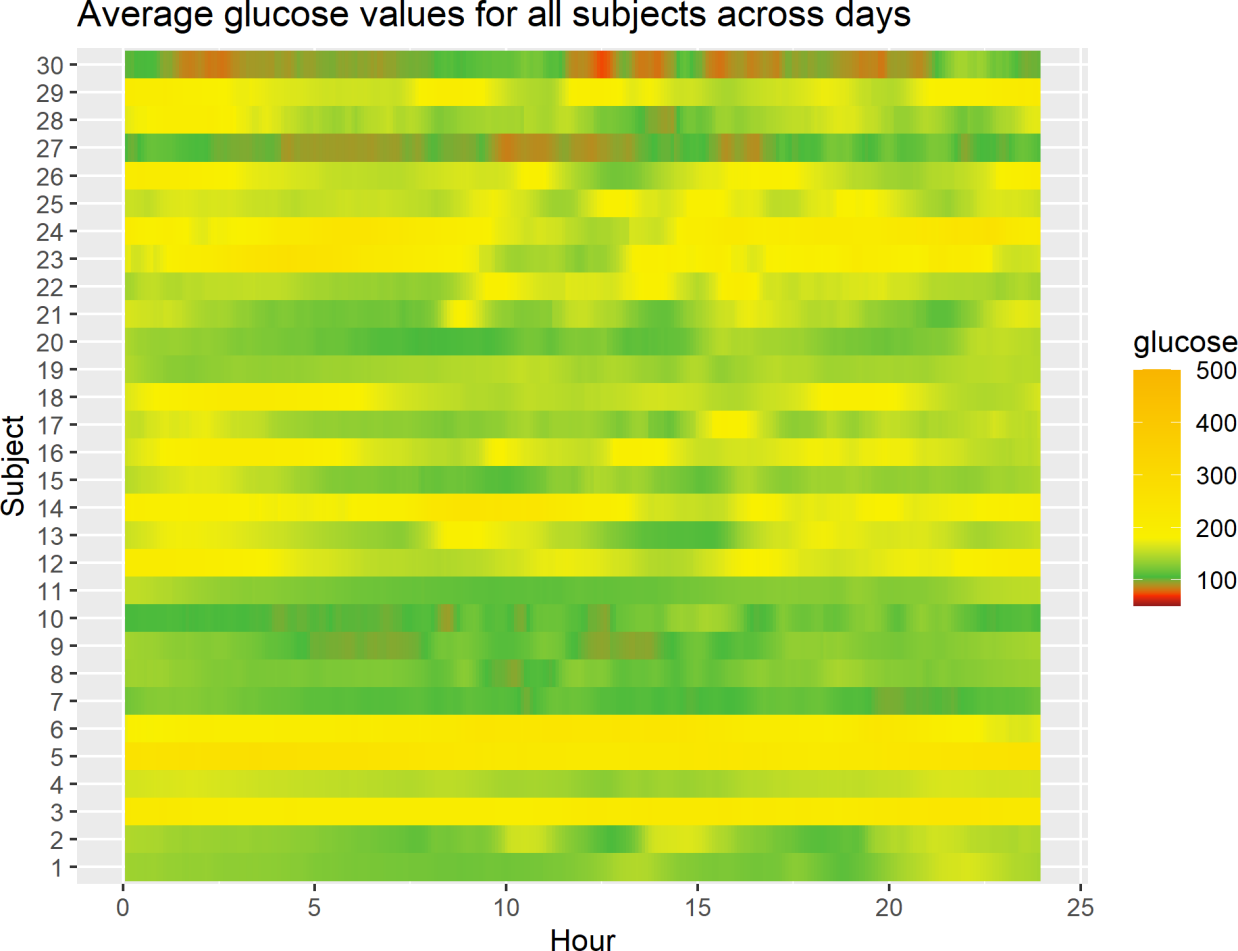
Lasagna plot of 30 subjects in the study. For any given subject, at any given time point in the 0- to 24-h range, the average glucose level over study days corresponding to that time point for that subject is displayed.

**Figure 1** displays a lasagna plot (27) of the average glucose level per subject over the 24-h period (i.e., for any given hour in the 0- to 24-h range, what is displayed is the average over the study days corresponding to that hour for that subject). One can observe some differences, both between subjects and within subjects, as highlighted by different colors in the heatmap. For example, some subjects have predominantly green profiles corresponding to glucose levels between ≈ 100 mg/dL and 180 mg/dL, whereas others have yellow profiles corresponding to glucose levels > 200 mg/dL. Also, one can see that some subjects have alternating patterns, for example periods of green and yellow and sometimes red, which correspond to low blood glucose levels, that is, roughly < 70 mg/dL. One limitation of the lasagna plot is that it reflects the average values only and not the variability of the measurements. Furthermore, it does not account for the fact that some participants may have more data than others, for example, because of the different length of time in the study or the different patterns of wearing the CGM devices. Finally, the lasagna plots do not demonstrate improving or worsening glycemic trends over time for the individual, rather the plots demonstrate glycemic averages for a given time of day.

**Figure 2:**
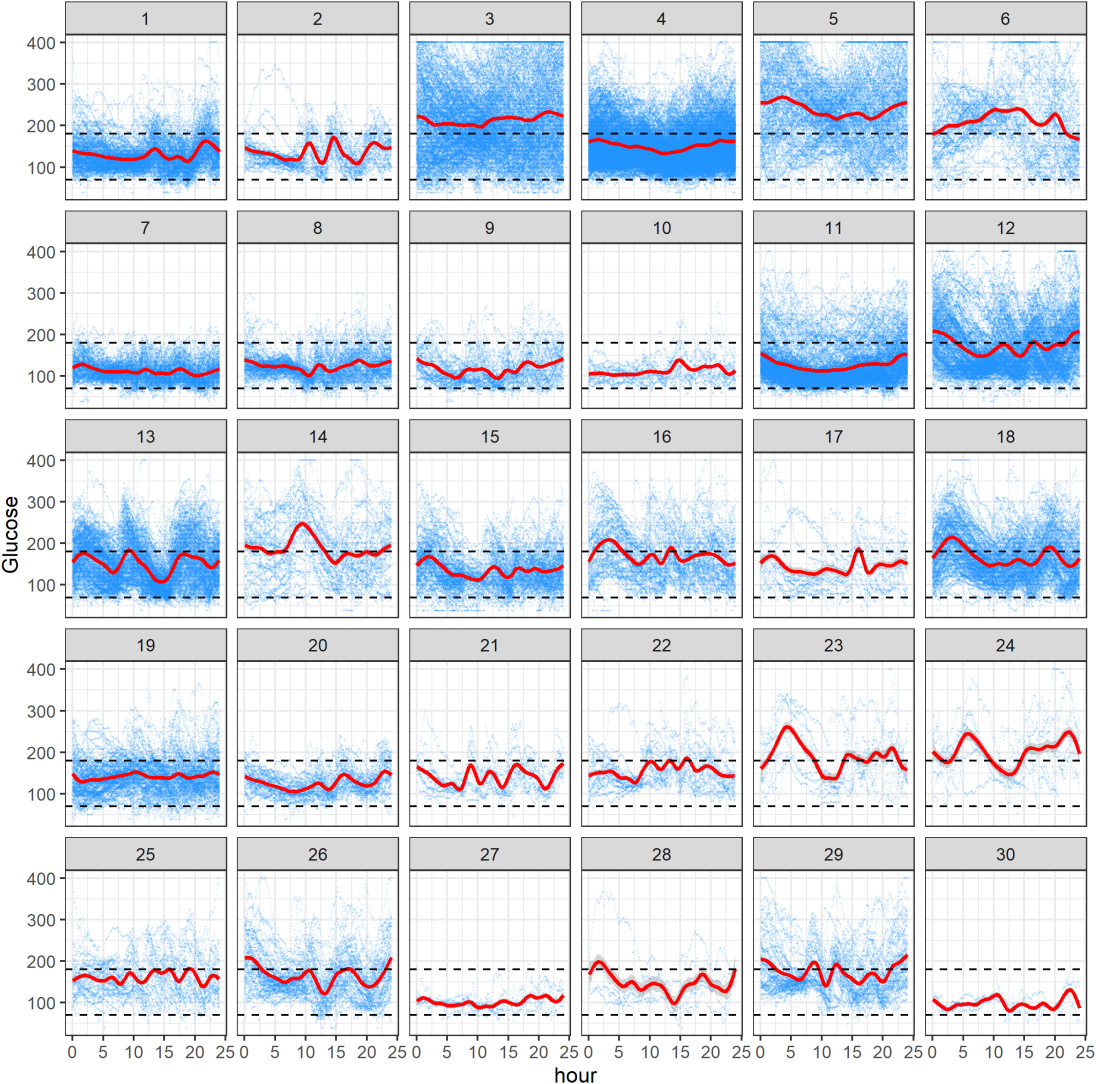
Ambulatory glucose profile (AGP) plots of 30 subjects in the study. Within each subject panel, the blue curves correspond to the raw CGM data acquired from the subject on different study days, and a red curve represents the mean AGP. The black dashed lines represent the range of 70 mg/dL to 180 mg/dL.

**Figure 2** displays all available CGM data for 30 subjects in the study. In each panel, the *x*-axis represents hours within the 0- to 24-h interval, and the *y*-axis refers to the glucose levels (mg/dL). Within a panel, the blue curves correspond to the raw CGM data acquired from a subject on different study days, and a red curve represents the mean AGP, obtained using smoothing techniques (28). The black dashed lines represent the target range of 70 mg/dL to 180 mg/dL (3.9 mmol/L–10 mmol/L). One can see from **Figure 2** that there is different amount of data per subject, reflecting the fact that some participants have been longer in the study or been wearing the CGM device for more days, and contributed more CGM data than others. For some subjects, there is evidence that the AGP is within the desired range, whereas for others it is crossing the upper limit of 180 mg/dL over the 24-h period. Notably, we observed some periodicity of AGPs over the day for some individuals. One should be mindful that the results depend on the amount of data, and the findings for an individual subject may change as more data for this subject are acquired during the study. Similarly to lasagna plots, these plots do not demonstrate improvement or worsening glycemic trends over time for the individual, rather they show glycemic averages for a given time of day.

**Figure 3:**
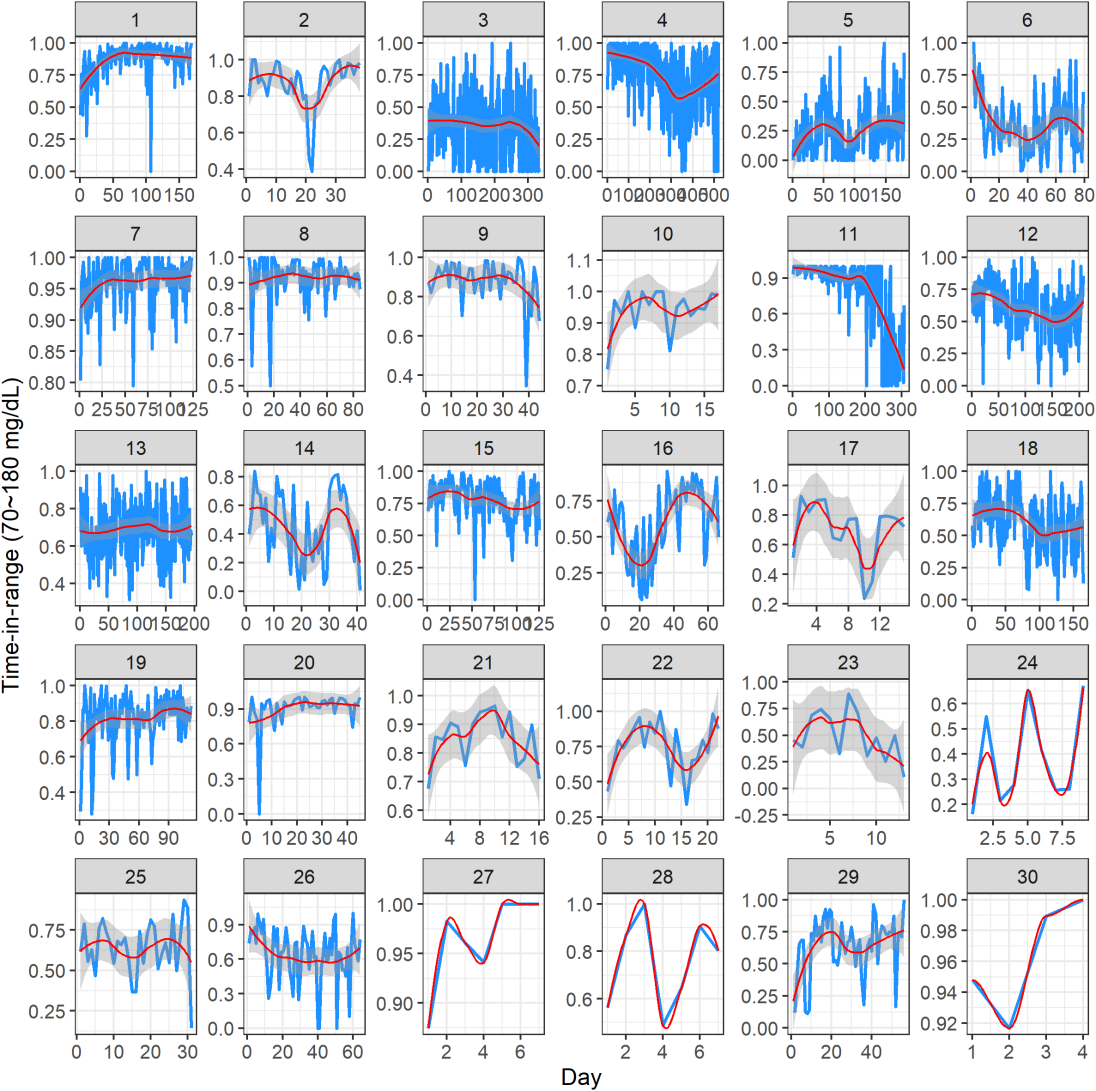
Dynamic time-in-range plots of 30 subjects in the study. Within each subject panel, the blue curve represents the individual time-in-range value on a given study day, the red curve is a modeled mean profile, and the gray area represents the uncertainty around the mean. The *x*-axis range is different across the subjects, as it reflects the different number of days of CGM data per subject. The *y*-axis range is displayed in a subject-specific manner because there was a substantial variation in the time-in-range values across the subjects.

**Figure 3** visualizes the TIR (i.e., percentage of CGM glucose readings in the target range of 70 mg/dL to 180 mg/dL) for 30 study participants. In each panel, the *x*-axis represents the study day, and the *y*-axis represents the TIR (proportion, measured on a scale of 0 to 1). Note that the *x*-axis range is different across the subjects, as it reflects the different number of days a given participant contributed CGM data in the study. The *y*-axis range is also displayed in a subject-specific manner, as there was a substantial variation in the TIR values across the subjects. Within a panel, the blue curve represents individual TIR values, the red curve is a modeled mean profile obtained using smoothing techniques, and the gray area quantifies uncertainty around the mean (28). The value of the TIR ≥ 70% is consistent with the clinically desirable target, as suggested by the American Diabetes Association (29, 30). From **Figure 3**, one can get some useful insights into the individual glycemic control over time. Some subjects have relatively stable patterns (e.g., subject 7), whereas for others there is evidence of fluctuation—increasing or decreasing the TIR values (e.g., subject 11)—indicating improved or worsened glycemic control over time. Clearly, the results depend on the amount of CGM data per subject, with more data enabling a more robust exploratory assessment. Importantly, these plots demonstrate the variability of the TIR across the duration of CGM use.

### 4.3 Clustering using glucodensities

**Figure 4:**
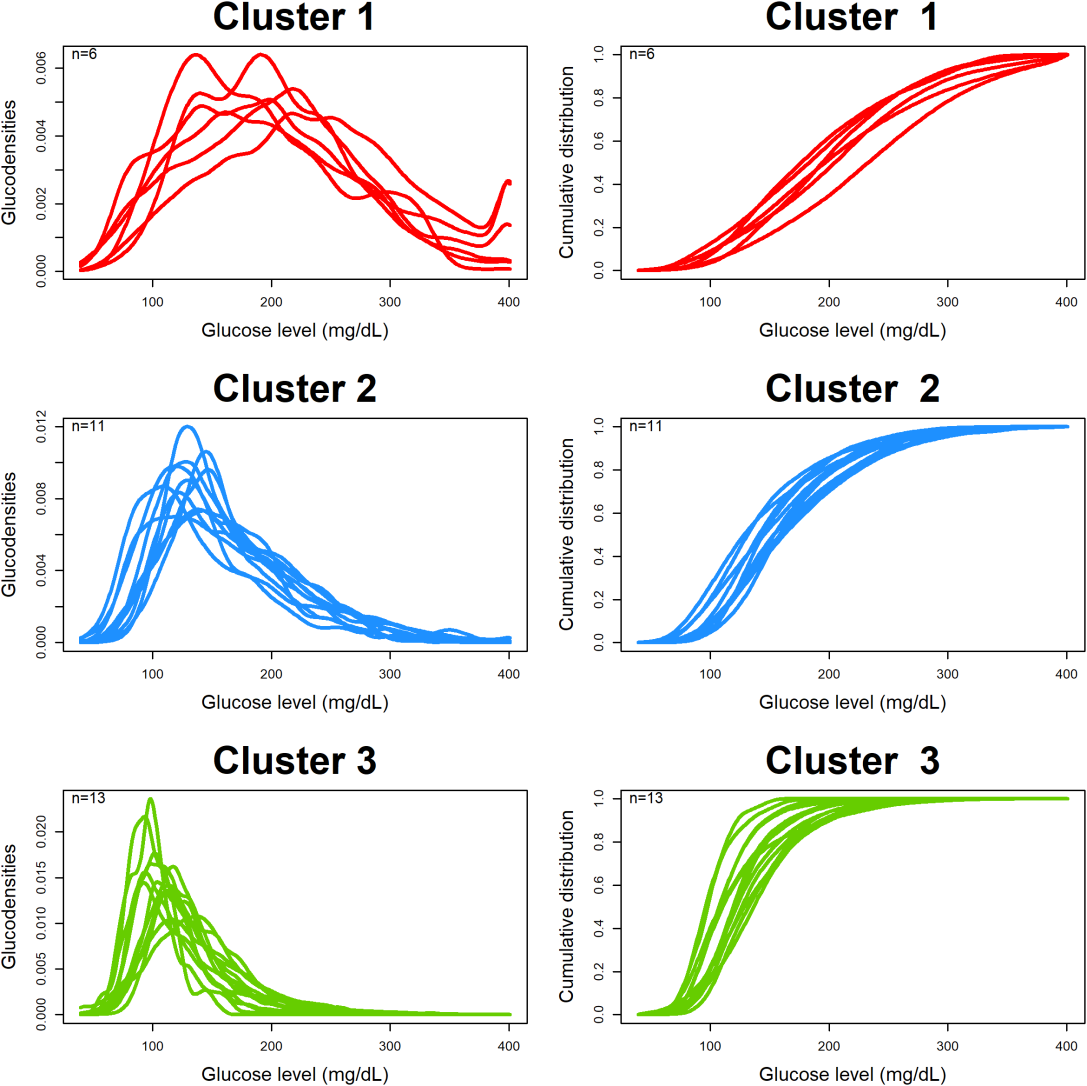
Clustering based on estimated glucodensities.

**Figure 4** shows the estimated and clustered glucodensities (left three panels) and the corresponding cumulative distribution functions (right three panels) for our dataset. Each glucodensity curve in a cluster corresponds to an individual subject, and they were constructed based on pooled CGM data from the individual. There are three identified clusters: red (six subjects), with the highest average and most variable levels of blood glucose; blue (11 subjects), with the somewhat better glycemic control; and, green (13 subjects), with the lowest average and least variable levels of blood glucose.

**Figure 5:**
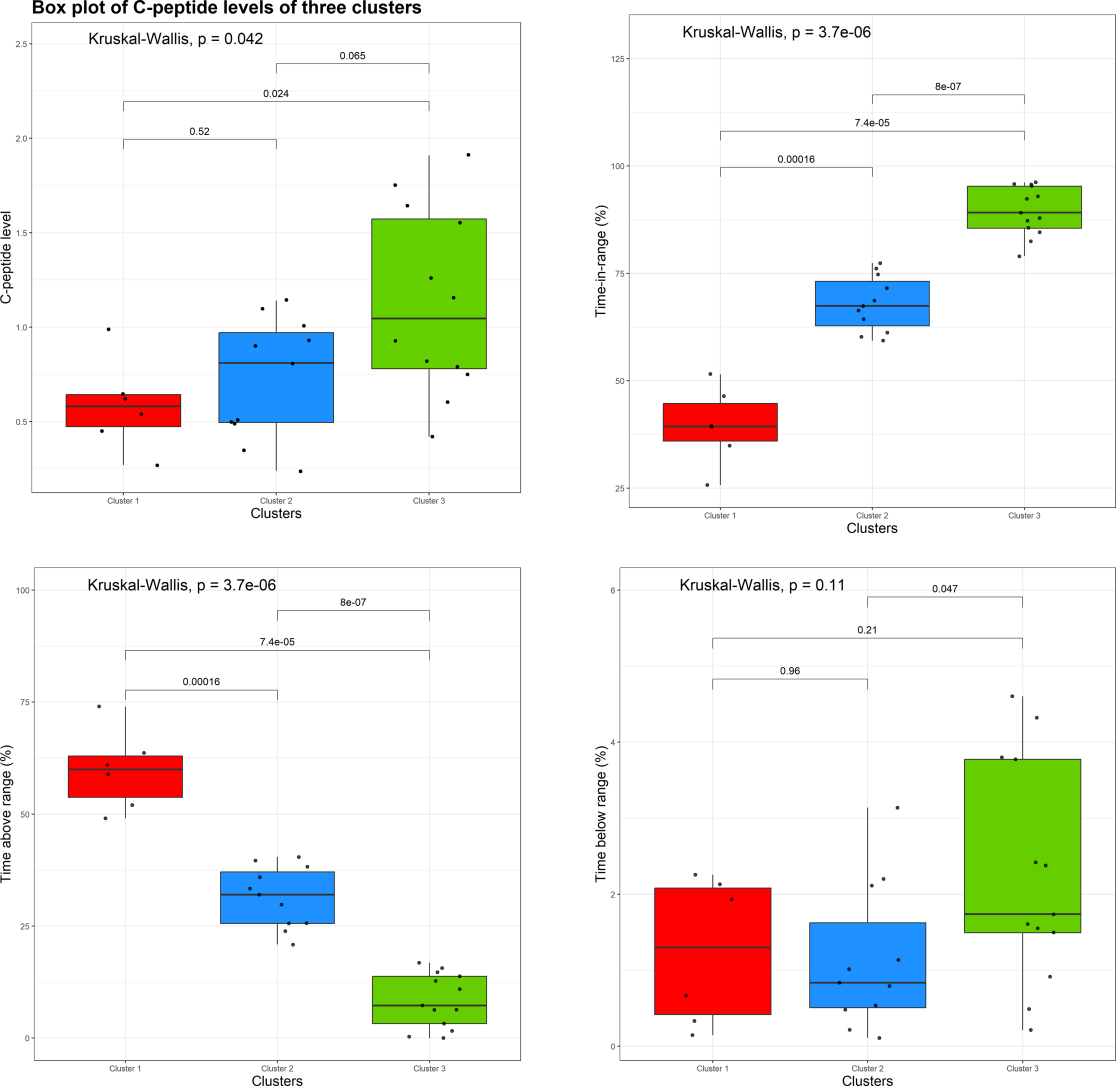
Boxplots of individual values and the results of a non-parametric analysis of variance (ANOVA) comparing three clusters with respect to the baseline C-peptide level (top left plot), time in range (top right plot), time above range (bottom left plot), and time below range (bottom right plot).

**Figure 5** shows boxplots of individual values and the results of non-parametric ANOVA comparing three clusters with respect to the baseline C-peptide levels (top left plot), TIR (the proportion of CGM measurements in the range of 70 mg/dL to 180 mg/dL; top right plot), time above range (the proportion of CGM measurements > 180 mg/dL; bottom left plot), and time below range (the proportion of CGM measurements< 70 mg/dL; bottom right plot).

From **Figure 5** (top left plot), there is evidence of an overall difference among the three clusters with respect to the baseline C-peptide levels (Kruskal–Wallis *p* = 0.042), with the difference being pronounced between clusters 1 and 3 (the red and green clusters, *p* = 0.024) and clusters 2 and 3 (the blue and green clusters, *p* = 0.065), but not between clusters 1 and 2 (the red and blue clusters, *p* = 0.52).

Also, one can see statically significant evidence of the difference between the three clusters with respect to the TIR (**Figure 5**, top right plot) and time above range (**Figure 5**, bottom left plot). In regard to the time below the range (**Figure 5**, bottom right plot), the overall difference among the three clusters is not statistically significant (Kruskal–Wallis *p* = 0.11); however, there is some evidence of difference between clusters 2 and 3 (blue and green clusters, *p* = 0.047).

## 5 Discussion

### 5.1 CGM data provides important information on T1D metabolism

CGM data collection is increasingly common in diabetes research (6, 21, 31–33). Recently, many statistical software packages have been developed to facilitate CGM data visualization and analysis. **Table 2** provides a summary of some of these packages. The *iglu* package (21) was useful for obtaining visual displays (e.g., lasagna plots) of individual CGM-derived metrics over a 24-h period. However, such plots reflect an overall measurement, but not the dynamics of individual glycemic parameters over time. We propose displaying relevant CGM metrics, such as the TIR versus the study day, to capture the dynamics of glycemic control and variability of the selected CGM parameters over time and simultaneously display the fact that some subjects contributed more CGM data in the study than others.

**Table 2:** R packages for analyzing CGM data.

Package	Reference	Usage	Remarks
*Iglu*	Broll et al. (21)	Exploratory data analysis	Comprehensive and user-friendly for visualization; limited input size
*CGManalyzer*	Zhang et al. (34)	Exploratory data analysis	Designed to read and organize the CGM data; no R Shiny available
*cgmanalysis*	Vigers et al. (35)	Exploratory data analysis	Designed to read and organize the CGM data; no R Shiny available
*biosensors.usc* (glucodensity)	Matabuena et al. (13)	Density estimation and cluster analysis	The first R shiny for modeling the CGM data; regression and clustering are available
*fda*	Ramsay et al. (36)	Functional data analysis	A standard package for analyzing functional data; preprocessing is required for the CGM data
*Refund*	Kokoszka and Reimherr (37)	Functional data analysis	A standard package for analyzing functional data; preprocessing is required for the CGM data
*fdANOVA*	Górecki and Smaga (38)	Functional data analysis	Designed to perform functional ANOVAs for intensive longitudinal data; useful for hypothesis testing with the CGM data

In the present work, we evaluated glucodensity (13) and performed a cluster analysis of estimated glucodensities based on the blinded CGM data of 30 participants from an ongoing study. Our analysis indicates that a clinically meaningful clustering using glucodensities is possible. We identified three clusters of patients with different shapes of glucodensities indicating different levels of blood glucose control. We found evidence of a statistically significant difference among the clusters with respect to some established biomarkers of T1D, such as residual pancreatic beta-cell function (C-peptide levels) at baseline, thereby indicating a potential grouping of patients with different severities of T1D. We also found evidence of cluster differences with respect to conventional CGM-derived parameters such as the TIR and time above range. Overall, the added value of statistical comparisons of the identified clusters using ANOVA, with respect to established biomarkers of pancreatic beta-cell function, is that it aids the clinical interpretation of the clusters. Although our findings are limited by the small sample size and the exploratory nature of the analysis, they are promising to characterize pancreatic function in stage 3 recent-onset T1D. Further analyses to validate these results are warranted once the study achieves the final database lock.

In our analysis, we focused only on the estimation and clustering of individual glucodensities. We did not attempt to estimate an average glucodensity curve for each cluster and the corresponding uncertainty. The estimation of “average glucodensity” from a sample of individual glucodensities and quantifying the associated uncertainty requires applying more advanced functional data analysis techniques such as the 2-Wasserstein distance and the Fréchet mean-of-the-density functions (13, 39). In addition, as the estimated glucodensities can be used as a basis for deriving CGM parameters (e.g., mean, standard deviation, coefficient of variation, TIR), another important problem is establishing a statistical agreement between empirical (i.e., obtained directly from the CGM data) and glucodensity-based CGM parameters. These additional questions require further careful investigation, and we defer them to future work.

### 5.2 Next step: quantifying treatment effects and treatment contrasts

An important objective of a randomized controlled trial in T1D is to compare treatment effects (IMP vs. placebo) by applying a statistical test on some clinically meaningful outcome measure. For this purpose, one can consider different CGM-derived metrics, such as the TIR, which is defined as either the percentage of CGM glucose values or the number of hours spent in the range of 70 mg/dL to 180 mg/dL during the measurement period (20, 40). One recent study found that 14 days of CGM data correlated well with 3 months of CGM data, and, within these, 14 days of at least 70% of CGM wear should provide robust data for deriving the TIR (41).

In the final analysis of our T1D clinical trial, for each trial participant, one can derive the baseline (pretreatment) TIR value and the TIR after 1 year of treatment, by taking their difference and then testing the significance of this difference (separately for the subjects in the IMP group and subjects in the placebo group) using a paired *t*-test. Furthermore, to assess treatment contrast (IMP vs. placebo), one can apply a two-sample *t*-test on the change from baseline values of the TIR. Alternatively, one can consider fitting an ANCOVA model as follows:


(3) $$TIR_{1,i}\;=\;\mu \;+\;\alpha \cdot \delta_{i}\;+\;\beta \cdot TIR_{0,i}\;+\;\varepsilon_{i},i=1,.\;.\;.,n,$$

where $TIR_{1,i}$ is the i th participant’s outcome after 1 year of treatment and $TIR_{0,i}$ is the similar value at baseline, $\delta_{i}\;=\;1$ (or 0), if the i th participant is assigned to the IMP (or the placebo), μ is the overall mean, α is the effect due to treatment (IMP), β is the effect due to TIR at baseline, and $\varepsilon_{i}$ ’s are independent, normally distributed measurement errors. By testing the hypothesis $H_{0}:\alpha \;=\;0$ we can address the following question: does the active treatment (IMP) have a significant effect on the TIR compared with placebo after 1 year of treatment?

Furthermore, extensions of the model (3) could be considered. In our T1D study example, the CGM assessments are made every 3 months during the 1 year of treatment. Although the primary interest is in the outcome at 12 months, the outcomes at earlier time points may be of interest as well. In this case, one could consider fitting a mixed-effects model with repeated measurements (MMRM) (42).

The TIR is one CGM-based outcome measure that can be analyzed using the approach described above. Other CGM-based outcome measures, such as the average glucose level, glucose measurement index, and glucose coefficient of variation (CV), can be similarly analyzed. To obtain a more complete assessment of the treatment effect, one can perform statistical analyses on multiple clinically important CGM-based measures and present the results (estimated treatment effects with corresponding confidence intervals and *p*-values) in one table. However, a limitation of such an approach is at least twofold. First, different CGM metrics are likely to be correlated, but the analyses of individual metrics do not account for such correlations. In fact, proper statistical adjustments for multiplicity are required to mitigate the risk of false-positive findings. Second, by deriving these different metrics, some important information may be lost. For example, the TIR does not account for the dynamic nature of the CGM data, nor the possibly unequal number of CGM data from individuals. This prompts the consideration of more complex CGM-based objects, such as glucodensities, for analyses.

Matabuena et al. (13) noted that glucodensity can be used “to establish if there are statistically significant differences between patients subjected to different interventions, for example, in a clinical trial.” Furthermore, Matabuena et al. (14, 15, 43) proposed methods and estimators for handling missing data and exploring their potential values in analyzing CGM data. In our considered setting, the main objects of interest are probability density functions, and functional data analysis techniques may be useful for statistical inference. For the i th individual in the sample, we assume there exists a “true” glucodensity $f_{0i}$ that describes the distribution of the individual’s glucose levels at baseline. It is plausible to assume that the individual glucodensity may change over time (e.g., due to treatment intervention received in the study, environmental factors, or disease progression). Let $f_{1i}$ denote the i th subject’s glucodensity after 1 year of treatment. An important question is: how could we measure “similarity” of $f_{0i}$ and $f_{1i}$ ? One could consider the 2-Wasserstein distance (13):


(4) $$d_{W^{2}}\left(f_{0i},f_{1i}\right)\;=\;\sqrt{\int_{0}^{1}{\left(F_{0i}^{-1}\left(s\right)\;-\;F_{1i}^{-1}\left(s\right)\right)}^{2}\text{d}s},$$

where $F_{0i}$ and $F_{1i}$ stand for the cumulative distribution functions of the glucodensities $f_{0i}$ and $f_{1i}$ . Metric (4) has some computational and modeling advantages, and it has a physical interpretation in the theory of optimal transport.

In practice, the functions $f_{0i}$ and $f_{1i}$ are unknown, but they can be consistently estimated from the CGM data of the i th individual by ${\hat{f}}_{0i}$ and ${\hat{f}}_{1i}$ ; see Equation (2). Then, the distance $d_{W^{2}}\left({\hat{f}}_{0i},{\hat{f}}_{1i}\right)$ can be computed to quantify the difference between the i th subject’s empirical glucodensities during pretreatment and posttreatment periods. Note that the pretreatment and posttreatment CGM data of an individual may be highly correlated. A statistical significance test may be considered for testing:


(5) $$H_{0i}:f_{0i}\;=\;f_{1i}\;\text{for}\;i\;=\;1,.\;.\;.,\;n.$$

Furthermore, let $\mu_{0}\left(s\right)$ denote the “average” glucodensity in the population of study subjects in the pretreatment period, and $\mu_{1}\left(s\right)$ denote a similar object for the period after 1 year of treatment. A research question is: are $\mu_{0}\left(s\right)$ and $\mu_{1}\left(s\right)$ different? In other words, one may be interested in testing $H_{0}:\;\mu_{1}\left(s\right)\;=\;\mu_{0}\left(s\right)$ based on the CGM data from the sample of study participants (separately for the IMP group and the placebo group). This problem can be viewed as a generalization of a paired *t*-test.

Taking a step further, for comparing treatment effects, one can consider the Wasserstein–Fréchet regression model using 1-year posttreatment glucodensity as the outcome, and treatment (IMP or placebo) and other clinically relevant covariates as predictors (39). This approach can be viewed as a generalization of an ANCOVA model (3) to functional outcomes. Relevant technical details, including estimation, statistical testing procedures, and asymptotic results, are beyond the scope of the current work. Petersen et al. (39) provided an example of applying functional regression analysis to post-intracerebral hemorrhage hematoma densities and found it promising in that context. We plan to address similar research problems for the CGM data and glucodensities in future work.

### 5.3 Conclusion and future work

This article outlined several research questions arising in the context of randomized clinical trials in T1D with CGM data and provided some preliminary ideas on how to tackle them in practice. Efficient visualization of the CGM data and cluster analysis using glucodensities are useful tools for exploratory analysis of the CGM data. Future work is needed to evaluate additional important issues. For instance, the estimand framework is increasingly useful in biopharmaceutical product development (44). Defining estimands based on glucodensities is a challenging but important and necessary next step for rigorously addressing clinical research questions concerning CGM data. Another important area of research is related to experimental design issues—sample size determination, choice of data collection time windows and time points, quantifying the amount of information from these data (e.g., Fisher’s information matrix)—and formulation of various optimal design problems. Finally, we think that the CGM data analysis provides an opportunity for a collaborative effort, integrating clinical, statistical, data science, and pharmacometrics expertise. Such a combination is increasingly viewed as being synergistic in solving complex problems in biomedical research (45, 46).

## Data availability statement

The datasets presented in this article are not readily available because of informed consent and confidentiality restrictions. The study is still ongoing. Requests to access the datasets should be directed to alex.sverdlov@novartis.com.

## Ethics statement

This study has been registered at https://classic.clinicaltrials.gov/ct2/show/NCT04129528. The studies were conducted in accordance with local legislation and institutional requirements. Written informed consent for participation in this study was provided by the participants’ legal guardians/next of kin.

## Author contributions

AG and MQ were involved in the design of the original study. OS and MQ conceptualized the statistical analysis plan. EC, MQ, DJ, and OS performed the statistical analysis. OS wrote the first draft of the manuscript. All authors contributed to the article and approved the submitted version.
